# A scoping review of different monitoring-technology devices in caring for older adults with cognitive impairment

**DOI:** 10.3389/fpubh.2023.1144636

**Published:** 2023-06-15

**Authors:** Hind Mohammed Asiri, Asia Mohammed Asiri, Hana Falah Alruwaili, Joseph Almazan

**Affiliations:** ^1^University of Bisha, Bisha, Saudi Arabia; ^2^King Khalid University, Abha, Saudi Arabia; ^3^Al Jouf University, Sakakah, Saudi Arabia; ^4^School of Medicine, Nazarbayev University, Astana, Kazakhstan

**Keywords:** technology devices, cognitive impairment, older adult, nursing care, caring

## Abstract

Various monitoring technologies are being developed to prevent potential complications among older adults with cognitive impairment and improve their cognitive function. This scoping review identified gaps in the development of monitoring-technology devices for cognitive health status and highlights areas that require further inquiry. This study used the Joanna Briggs Institute (JBI) and the PRISMA extension for the checklist for scoping reviews using the eligibility criteria recommended by Population, Concept, and Context (PCC) framework. The study population included adults aged 65 years and above, while the concept and context are monitoring-technology devices utilized in detecting and caring for an older adult with cognitive impairment. Three electronic databases (Medline, Scopus, and Web of Science) were searched, and a total of 21 articles met the selection criteria. Several innovative technology-based devices for screening, assessing, detecting, and monitoring the interventions for older adult cognitive impairment and for family caregivers to ensure the continuity of care were established. Monitoring devices are useful in promoting older adult safety, improving their quality of life by enabling them to live independently for a longer period, and improving their mental wellbeing to help reduce the burden on caregivers by providing them with information concerning the activities of older adults. Moreover, studies have shown that older adults and their caregivers can learn to use these devices effectively and comfortably with proper education and training. The results of this study provide crucial insights into innovative technologies that can be used to assess cognitive health among older adults, which could substantially improve their mental health, and this baseline information can be used for supporting public health policy and enhancing their quality of life.

## 1. Introduction

The population of older adults has been substantially increasing worldwide (1). In 2019, over one billion older people were reported to be aged 65 years and above, and by 2030, this number is estimated to increase to 1.4 billion (2). In Asia, particularly in South and Southeast Asia, Latin America, and the Caribbean, a rapid increase in the aging population has been observed, which is evidenced by an increase in the percentage of older people from 6% in 1990 to 11% in 2019 in East and Southeast Asia, while it was 5% in 1990 and 9% in 2019 in both Latin America and the Caribbean (1, 3). Progressive decline in cognitive domains is a common phenomenon associated with aging; however, a substantial decline can also lead to cognitive impairment (CI). Patel and Singh (4) pointed out that older adults are more susceptible to CI, other neurogenic health conditions, and symptoms associated with aging, which can negatively affect their activities of daily living and quality of life (5).

Monitoring-technology devices are particularly important for older adults to assess and detect both their cognitive and functional statuses, as they can help them to evaluate their overall health condition, maintain their independence, and improve their quality of life (4–18). Currently, several technology devices are being developed to monitor the health problems of older adults, including CI (6–9). Some of the currently available monitoring technologies are telerehabilitation (7), smart aging systems (8), monitoring systems (9), wearable sensor-based sensors (10), cognitive and training apps (11), virtual cognitive health (12), intelligent home monitoring systems (13), technology-enhanced multi-domain (14), and high-performance eye-tracking technology (16). Specifically, assistive technology devices, such as wearable sensors for monitoring sleep, utility, usage, motion, and presence, have also been used as home monitoring systems for supporting older adults with CI (12). Other researchers designed psychosocial interventions based on the data obtained from the assistive technologies used to enhance the cognitive functions of the patients, which further enabled clinicians to identify interventions appropriate for them (13). A recent study described that the use of a wearable inertial sensor as an alternative for screening CI indicated that cognitive impairment could be observed in daily walking activity, particularly by reducing the walking speed, length of stride, and stability (10). In addition to the use of wearable type of sensors, transforming the homes of older adults with dementia into smart homes for activity recognition and anomaly detection has been carried out by previous researchers (6, 8). Bonnechère et al. (11) reported that, other than sensors, cognitive mobile games (CMG) ca also be used for enhancing the cognitive performance of older adults. Internet-delivered lifestyle interventions were also effective in delaying the progress of Alzheimer's disease (12). Another approach for improving their cognitive performance is by implementing the Ability Program, a “Technology-enhanced version of the home-based usual care program” (TAU). This program can assess the individual's quality of life, thus making the work of healthcare professionals during the rehabilitation phase easier (14).

Additionally, high-performance eye-tracking technology that records the gaze points of the patients while watching task movies and pictures is a novel method of diagnosing patients with CI and dementia (15). The use of the near-infrared spectroscopy (NIRS) system as an alternative imaging system is also useful in analyzing brain activation in cognitive disorders (16). Machine learning techniques such as “Gradient Boosting Machine” (GBM), an advanced learning algorithm that classifies the collected data from keystroke patterns and smartwatches attached to older adults, can categorize the severity level of CI with more than 94% accuracy, which could subsequently help in the prediction of CI (17). The advent of recent technologies can be considered a huge milestone in improving the mental health wellness of older adults to enable them to live independently and achieve an optimum level of functioning. It is also worth noting that these devices can greatly enhance older adults' safety, improve their quality of life and wellbeing, achieve their optimum functions, and reduce the burden on caregivers.

Meanwhile, there is a growing interest in the academic research area toward the prevalence of CI among older adults and the potential benefits provided by monitoring-technology devices for improving their daily activities, social behaviors, and mental wellbeing (16–20), which further promote safety and mobility. It is worth considering the fact that caring for older adults with CI has remained a challenge for healthcare professionals in recent years (18, 19). For example, one recent study reported that nurses face difficulties while performing physical assessments for older adults with CI compared to when performing them for other patients due to the former's responsive behaviors (e.g., restlessness, yelling, and hitting), which takes more time and effort to assess (18). The changes in behavior, the use of several medications, and the occurrence of comorbidities in older adults with CI have made the delivery of medical and nursing care even more complicated (20). Moreover, the implementation of technology in the care of older adults with CI required technical knowledge throughout its implementation; however, the fact that older adults faced difficulties in accepting and getting used to the technology was also reported (18). Despite these challenges, several studies have reported that the use of technology-assisted devices play a crucial role in caring for older adults with CI, as they provide safety, security, medication management, and monitoring of daily activities, which contributed to improved care and quality of life (7, 21–23). Although several studies reported the importance of these devices in healthcare, there is no review article of sufficient scope that outlines technology devices and their care management.

Therefore, this study identifies the current research evidence on monitoring-technology devices for older adults with CI, including different devices, their effectiveness, acceptability, and limitations. The results are crucial for healthcare professionals and policymakers in making healthcare decisions concerning the use of these devices in both clinical and community settings.

## 2. Methods

### 2.1. Design

**Figure 1:**
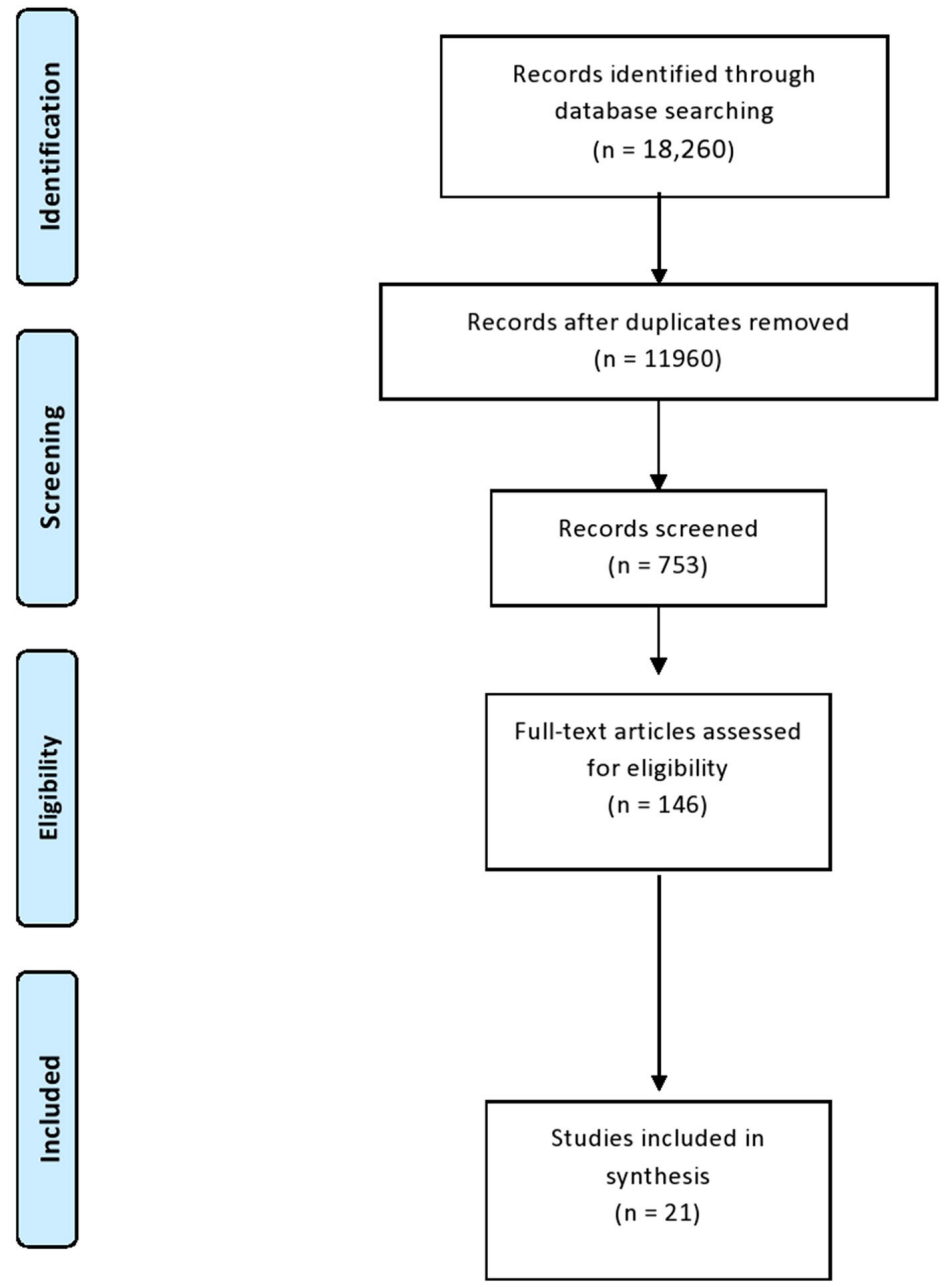
Search flow process.

This scoping review used the “Joanna Briggs Institute” (JBI) (24) and the “PRISMA extension for scoping reviews” (PRIMSA-ScR) (25) (Figure 1). The eligibility criteria were defined using the Population, Concept, and Context (PCC) framework (24). The study population for this review comprised older adults who are 65 years old and above. Studies involving the utilization of monitoring-technology devices in caring for older adults with cognitive impairment were selected. Specifically, full-text manuscripts concerning screening devices related to cognitive impairment and published in the English language were included. The exclusion criteria were as follows: the presence of non-cognitive impairment diseases, such as falls, conferences, book chapters, and unpublished or gray literature.

### 2.2. Search outcomes

Three electronic databases (Scopus, Web of Science, and Medline) were chosen for the selection of review articles. All the articles have a title, an abstract, and keywords that conform to the following Boolean expression: (Cognitive impairment OR Cognitive Dysfunction AND cognitive screening OR cognitive test OR “memory screening” OR “memory test” AND monitoring-technology devices OR Artificial system devices OR multi-modal neuroimaging technique OR sensor devices OR technology application OR sensor technology AND older adults OR Old age).

### 2.3. Selection and article analysis

First, all authors assessed the titles and abstracts of 18,260 articles for relevance, and those not meeting the inclusion and exclusion criteria were removed. The authors removed 6,300 duplicates, references without abstracts, and articles that were not written in English. The authors then assessed the full text of the remaining articles against the outlined inclusion and exclusion criteria, and the final list of the articles to be considered for the review was prepared. Throughout the entire selection process, each article was analyzed by three authors, and any disagreement between the authors was discussed and resolved by consensus. A total of 21 articles met the inclusion criteria (Figure 1).

## 3. Results

A total of 21 articles were included in this study and were published between the years 2008 and 2022 (Table 1). The majority of the included studies were conducted in the United States (12, 26–28, 30) (*n* = 5), followed by China (*n* = 5) (10, 16, 27, 34, 37), Italy (*n* = 3) (14, 29, 33), and South Korea (*n* = 2) (17, 34). One article each was conducted in Greece (*n* = 1) (13); the United Kingdom (11); Japan (15); Thailand (32); Sweden (6); and Australia (36). Specifically, 14 studies developed a system/technology that recruited various types of respondents. Specifically, six studies focused on older adults with mild CI, AD, and dementia (10, 13, 14, 16, 29, 34), two articles focused on comparing healthy older adults and those with mild CI (15, 30), five studies focused on older adults in general (11, 12, 17, 28, 32), one study included college students and certified professional controllers as respondents for the optical brain-monitoring technology (37), and the remaining three studies did not specify any respondent (4, 26, 37).

**Table 1:** Studies included in the scoping review.

**References/country**	**Functional variable (operationalization) Device**	**Aims**	**Data collection and analysis**	**Findings**
Ayaz et al. (26)/USA	Functional near infrared spectroscopy (fNIRS)	To determine the reliability of fNIRS in monitoring brain skill acquisition and task performance	The study described various studies that used fNIRS	Results revealed that a portable, wireless fNIRS system is reliable in monitoring the cognitive brain and working memory training
Ayaz et al. (27)/USA	Functional near infrared spectroscopy (fNIRS) Continuous wave fNIRS system	To assess the: 1) “Mental workload of operators performing standardized (n-back) and complex cognitive tasks (air traffic control—ATC)” 2) Development of “complex cognitive and visuomotor tasks (piloting Unmanned Air Vehicles—UAV)”	Respondents were asked to complete two tasks: 1) A “visual identity nback and two types of ATC tasks.” The n-back task was a standardized working memory and attention task with four incremental levels of difficulty 2) “Normalization by linear modeling. A personalized model was developed based on the fNIR responses for the standardized task to quantify the responses measured during ATC tasks”	fNIRS measures are sensitive to mental task load and cognitive and visuomotor systems, and can monitor hemodynamic changes that are related cognitive workload
Bonnechère et al. (11)/United Kingdom	Applications that train the brain in the form of cognitive games	Analyze the game scores and the processing speed of adults aged 60–80 years and above	The researchers first identified the number of days needed in order to perform all sessions, and the first session of brain training was analyzed in order to determine the influence of age on their initial score of the cognitive games. The difficulty level of the game was increased to determine the processing speed of the adults	In terms of the initial CMG scores, it decreases along with increasing age of participants When the difficulty of the game was increased, it revealed that in *word pairs* game, all age groups decreased in processing speed. In *square numbers* and the other remaining games, the processing speed increased but the score while the processing speed increased is observable among younger participants
Bott et al. (12)/USA	None	To assess the baseline characteristics of the participants in the VC health study	Participants underwent a baseline survey and then attended the Virtual Cognitive Health program. Online assessment was done at 12, 24, and 52 weeks	At baseline, patients were found to have mild levels of anxiety and depression and minor subjective cognitive impairment
Chan et al. (28)/USA	Training with the use of tablet computers	The findings of the study indicated that there was a significant increase in cognitive performance and episodic memory among participants who underwent iPad training compared to the other groups	An experimental design comprised different groups. One group of patients underwent training using iPad intervention; another group engaged in passive tasks with less new learning; and a final group had regular social interaction but no active skill acquisition. Then cognitive and psychological pretests and posttests were compared	Results revealed increased improvement for cognitive performance and episodic memory using iPad training compared to other groups
Chimamiwa et al. (6)/Sweden	None	To determine whether smart homes are adequate among older adults with dementia	The researchers surveyed the literature and existing works on detecting anomalies and activity recognition for Older adults with dementia (OAwDs). They also pointed out its transition within the stages of dementia and then presented some challenges that are essential to be addressed	The researchers presented the capabilities of smart homes and the stages of dementia, and justified why habit recognition in smart homes is essential for adults with dementia
Cottelli et al. (29)/Italy	Repetitive transcranial magnetic stimulation	Assessed the effect of repetitive transcranial magnetic stimulation applied to the dorso-lateral prefrontal cortex on picture naming in 24 probable AD patients with different degrees of cognitive decline	Experimental design with two groups: 1) mild and 2) moderate to severe cognitive impairment based on Mini-Mental State Examination •Groups were instructed to sit in front of a computer and were tasked to name the picture as quickly as possible while a high-frequency repetitive transcranial magnetic stimulation was attached to the left and right dorsolateral prefrontal cortex (dlPFC)	Results found that after repetitive transcranial magnetic stimulation, the performance of patients suffering from severe cognitive decline improved in naming the object
Dorociak et al. (30)/USA	None	To evaluate the convergent validity of a web-based cognitive assessment tool and the implementation of the Survey for Memory, Attention, and Reaction Time (SMART) for older adults	Participants were grouped into two: 1) cognitively intact and 2) diagnosed with mild cognitive impairment, and completed four cognitive tasks such as visual memory task, trail making test, and Stroop color word interference task using a computer tablet at home. They were assessed using neuropsychological tests and other functional assessment measures	Results showed the group with mild cognitive impairment had lower neuropsychological test scores compared to the cognitively intact group. Moreover, time to complete each test item was significantly higher for the MCI group SMART tasks were found to be strongly related to the status of cognition, and the outcome measurement illustrated fair to excellent test–retest reliability
Hossain et al. (17)/South Korea	None	To develop a machine learning model to detect and differentiate the cognitive impairment categories of severe, moderate, mild, and normal by analyzing neurophysical and physical data	Keystroke and smartwatches were used to identify the keystroke patterns and the physical data of the older adults. They also underwent a session to answer a questionnaire where the MMSE score was produced in order to indicate cognitive impairment	Results showed that the model was able to detect varying levels of cognitive impairment (normal, mild, moderate, and severe) CI with accuracy of 94.8%
Niu et al. (31)/China	Near-infrared spectroscopy (NIRS) system	To study the spatiotemporal dynamics of oxygenate hemoglobin during a working memory task among patients with MCI and healthy controls	Participants underwent four neuropsychological tests to assess the general mental status and other cognitive domains A blocked periodic design consisting of alternating 0 and 1 back tasks was utilized during the WM task Researchers used ETG-400 optical topography system in order to measure the concentration the changes in oxy-Hb and deoxy-Hb	During the 0 and 1 back test, the healthy individuals performed better than the MCI patients Moreover, MCI individuals illustrated a decreased activation in the left frontal, right superior frontal, and left temporal lobes •Also, there was changed frontal and temporal processing during working memory tasks in the MCI patients
Lazarou et al. (13)/Greece	Novel and intelligent home monitoring system	To design personalized interventions based on system feedback and clinical observations for improving cognitive function and health-related quality of life	Intervention design Participants underwent a 7-month psychological intervention based on cognitive and behavioral limitations (neuropsychological assessment and the system's visualizations were introduced) The monitoring system was installed in the home of the participants in order assist the independent living of people with dementia	The system generated the required tools for clinicians to support and foster the quality of life of participants through the use of this assistive technology The installation of the system improved the cognitive function and many behavioral aspects of the participants. It was also able to reduce anxiety and improve the performance of the participants cognitively
Muangprathub et al. (32)/Thailand	Tracking system with the use of mobile and wearable sensors	To develop a tracking system using the integration of multiple technologies combined with machine learning to obtain a new older-adult tracking system that covers aspects of activity tracking, geolocation, and personal information in an indoor and an outdoor environment	The system composed of two parts: the wearable device and the web applications, commands on wearable devices, and commands on the server as the software of the system. The participants wear this device in the right side of their hip The tracking system was tested among older adults and was matched with their data who have used the device	The tracking device was able to detect the movement of the older adult, and it provided real time monitoring and alerts. The system was also found to display information in spatial format, and the participants could use the device to send messages in emergencies
Cammisulli et al. (33)/Italy	SENIOR (SystEm of Nudge theory based ICT applications for OldeR) citizens	The SENIOR project was designed to adopt an advanced information and communication technology coaching system able to collect and integrate holistic data from older adults	Randomized controlled trial involving 200 older adults with CI and evaluated by the MethoTelemed, a framework methodology to assess the effectiveness of telemedicine applications	The SENIOR project develop tools and protocols that can be useful in the field of preventive medicine, and clinical efficiency through data analysis is evaluated
Lim et al. (34)/South Korea	Internet of Things (IoT) platform	To evaluate the activities of daily living in both healthy older adults and cognitively impaired older adults using IoT devices and network technologies	Participants with MCI and dementia and cognitively healthy individuals performed a total of 13 experimental ADL tasks, and the total time in order to complete the task was evaluated	The group of patients with dementia had the lowest success proportion compared to the MCI patients and the healthy individuals, and their group required more time to complete the tasks Moreover, there was a relationship between severity of the impairment with the performance of experimental ADL tasks, and the assessment of ADLs using IoTs allowed for an accurate evaluation of the functional level of the patients
Karakostas et al. (35)/Greece	Sensor-based system with the use of remote web technologies	To assess cognition, emotion, and quality of life of patients with mild CI	The monitoring sensor-based system attached in their homes assessed participant's ADL performance	Based on the study, the preliminary results were positive, indicating improvement in personal hygiene and in the number of sleeping hours at nighttime due to fewer interruptions, ADLs, lower usage of TV, better management of personal problems, and decline of anxiety and depressive symptoms levels
Realdon et al. (14)/Italy	None	To test home-based multidimensional program technology in enhancing the continuum of care for mild CI and dementia patients	Patients with MCI and AD were randomized and grouped, and underwent two separate rehabilitation sessions. One measured vital parameters at home through the usual care treatment program and the other through the ability program Assessment was done before the start, at the end of the session (8 months after), and a follow-up (12 months after the baseline)	The trial was ongoing, but the researchers of the study hoped that the Ability program could be as promising as a usual home procedure in assisting patients with MCI and AD
Oyama et al. (15)/Japan	High-Performance Eye-Tracking Technology Eye-tracking system which monitors the gaze points of the individual through the use of camera and infrared light sources	To develop a novel brief and practical cognitive assessment tool utilizing an eye-tracking technology with mild CI	An assessment using the eye-tracking system was used which allowed the participants to watch a sequence of short movies and pictures while their gaze points were recorded by the eye-tracking device The cognitive scores were measured in healthy individuals as controls, patients with MCI, and those with dementia Participants underwent both MMSE and eye-tracking-based cognitive assessment, and the correlation was evaluated	The eye tracking-based cognitive scores showed a good association with the scores from the neuropsychological tests It also showed an accurate discrimination among patients with dementia and MCI
Oatley et al. (36)/Australia	The smart textile prototype comprises games and data logging systems including the physical computing, components in music playing, and simple interaction	To produce a hardware system assessing the behavior of patients with dementia and mild CI	The behavioral patterns were recorded using the data logging system, and the textile was placed in the laps of the resident to play, generating a behavioral profile through the gamification of cognitive tests	The textile was able to function well with simple interaction, and its simple input/output technology can be easily installed under changing textile layers. Personalization of the textile was easy people new to the device
Patel et al. (4)/US	AI technologies	To highlight emerging AI technologies used in the caring and management of patients with neurological disorders to evaluate improvements in overall functional outcomes of patients	The researchers described various forms of AI used and those that are available to use, including previous studies, outcomes, benefits, and limitations	There were various limitations of artificial intelligence, however, studies nationwide concluded that this type of technology had the capacity to enhance the prognosis of neurological disorders since it aids in analyzing data in the medical field, which would be helpful in preventing, diagnosing, monitoring, and developing new protocols
Xie et al. (10)/China	Wearable sensors	To characterize gait disorders in patients with amnestic mild cognitive impairment and to determine the association between the performance of the gait function and cognition	Patients with amnestic mild cognitive impairment and healthy controls were assessed neuropsychologically. The gait data was collected through a wearable device	The patients with aMCI had increased stride time variability, and reduced walking velocity and stride length compared with the healthy individuals The memory and executive function were associated with stride length and walking velocity, while stride variability was negatively correlated with attention, memory, and executive function
Zhou et al. (37)/China	Internet of Things System (IOT) Diagnosis of Alzheimer's disease (AD) based on the Internet of Things Systems	To evaluate IOT in diagnosing patients with suspected Alzheimer's disease	Experimental design with the use of dimensionality reduction, the formula of classification problem (in a variety of solutions to merge the class), and machine learning methods	The new non-invasive technology screening test interactions, and a new non-invasive early diagnosis method enables faster, improve testing and treatment of patients suspected of having AD

The experimental design was employed in most of the sampled articles using randomized controlled trials and comparing groups of participants including data from developed devices. Five of the 21 sampled articles were systematic and integrative review studies that summarized the current evidence on the advantages of artificial intelligence (AI), smart home installations, the use of functional near-infrared spectroscopy, and smartwatch applications in aiding patients with CI (4, 6, 27, 33). The sampled articles covered various aspects of technology and CI and are presented under three subtopics.

### 3.1. Novel method for the assessment and screening of cognitive impairment

Previous research has shown that a high-performance eye-tracking technology is a good diagnostic procedure for detecting mild CI and dementia. It was demonstrated that, when the respondents were asked to view the short clips of movies and images, their eye-tracking score correlated well with the scores of neuropsychological tests (15). Wearable sensors (which are used for walking) may also be an alternative approach for screening CI, as it has been found that reduced speed, stride length, and stability when walking are more common among mild CI patients (10). A new assessment method called Survey for Memory, Attention, and Reaction Time (SMART) was found to show fair to excellent test–retest reliability, and it can be used to monitor cognitively healthy and older adults with mild CI (30). In another study, mobile apps were utilized for brain training, and it was found that the cognitive mobile score of patients decreased as age increased (11).

In a separate study, another approach was used to determine the severity of CI, that is, by measuring the performance of patients with dementia and mild CI in activities of daily living (ADL), wherein the researchers reported that the group comprising dementia patients showed the lowest proportion of success in ADL (38). Non-invasive near-infrared spectroscopy was also found to be a useful tool for assessing the activation of the brain in cognitive disorders, since a previous study showed that those with mild CI have reduced activation in their left temporal, left frontal, and right superior frontal lobes (16). A study also reported that keystroke patterns and physical activity data of a person with CI could be used to differentiate cognitive severity levels through a proposed Gradient Boosting Machine (GBM), an advanced learning algorithm (17). The same concept was used in a study where Alzheimer's disease (AD) was diagnosed through the Internet of Things (IoT) monitoring system and a deep learning classification model (37).

### 3.2. Use of technologies in monitoring systems

Concerning the usage of technology in monitoring, one study in Thailand developed a tracking system with the use of multiple technologies that can track activity and information in indoor and outdoor environments of older adults in real-time and was able to send alerts displayed in a spatial format, and it can also act as a messaging device in case of emergency (32). The development of a sensor-based system was also able to improve sleep and support the daily living of people with AD (35). A concept article proposing a prototype of a smart textile was created to monitor the behavior of patients, and according to the researchers, data logging systems can be installed (including music and games) to keep the patient entertained (36). The installation of assistive technology devices, such as sensors, in the homes of older adults with dementia was found to be effective in providing sufficient data that could be used for planning interventions suitable for the patient and for supporting clinicians (13). In another study, repetitive transcranial magnetic stimulation in the dorsolateral prefrontal cortex was able to restore damaged cognitive function among AD patients (29).

### 3.3. Programs and interventions designed for improving the lives of older adults with cognitive impairment

A previous study has shown that the Ability Program, with an ongoing trial status, involves enhancing the effectiveness of a home care program with the integration of technology. This intervention was applied continuously for a period of 6 weeks, and it is anticipated that the program can improve the cognitive function of older adults with mild AD and mild CI (14). Training older adults using tablet computers was also found to enhance their cognitive domain when compared to performing social or non-challenging activities (28). Participants with memory impairment who enrolled in the intervention for a healthy diet, cognition, exercise, and sleep revealed that remote intervention could be a suitable solution for delaying the progress of AD (12).

## 4. Discussion

The studies included in this scoping review were analyzed to identify which technology is most frequently studied and developed to improve the wellbeing of older adults with CI (11–30, 32–38). Sensor-based technologies were the most common type of technology studied and were developed mainly for screening and monitoring CI (6, 8, 32, 35, 36). On the other hand, virtual apps, such as cognitive mobile games, were found to be the least explored aspect based on the sampled studies (11). The presumption is that sensors are the key factors in acquiring specific and relevant information from older adults with CI even on a completely remote setup. Various studies have provided evidence that sensors, as the main feature of a monitoring device, successfully provided a wide range of data concerning older adults with CI, such as their gait speed, gait stability, gait stride, geolocation, behavioral change, anomaly detection, and data regarding other physical activities, suggesting that it could be one of the most important areas to explore by researchers (10, 17, 32, 35).

The development of sensors with advanced functions is an important area of research because the promising results of these technologies are expected to highly contribute support not only to patients with cognitive impairment but also to healthcare professionals. With a rapid rise in the percentage of the older population becoming affected by the problem of cognitive impairment, sensors became the most common solution for helping older adults to live independently (6, 8, 32, 35, 36). When the sampled articles were also analyzed in terms of their purpose, it was found that the target individuals for the monitoring-technology devices were mainly older adults with either healthy cognitive function or CI (4). Few of the assistive technologies developed were installed in the residences of older adults, thus transforming them into smart homes, and some created wearable sensors for older adults, while a large proportion of technologies mentioned in the sampled articles did not specify the target population (6, 32). Despite the development of various technologies, such as the intelligent diagnosis of AD, brief cognitive assessment using high-performance tracking technology, and wired and wireless functional near-infrared spectroscopy system for the evaluation of brain activation and repetitive transcranial magnetic stimulation, a research gap was found when these technologies were applied in the actual healthcare institution for offering an alternative way of assessing and detecting CI (16, 26). In the past, technological devices mainly focused on discovering novel methods for detecting anomalies in the cognitive functions of older adults. Nevertheless, the sampled articles in this review also showed that assistive technologies and interventions were created to increase the ability of older adults with CI to live independently (6, 8, 32, 35, 36).

In addition, the process involved in the development of monitoring-technology devices for CI is essentially important, as it can lead to the creation of effective technology tools for detecting and monitoring older adults' cognitive decline. By accurately measuring cognitive functions over time, these devices can help in identifying changes in cognition that may be indicative of the underlying health conditions. For example, in the experiment of Ayaz et al. (27), the participant's brain was monitored to measure the cerebral blood oxygenation changes by using the functional near-infrared spectroscopy (fNIRS) continuous wave system. During this study, the fNIRS raw data were processed using several algorithms to remove noise and convert the signal into meaningful measures of neural activity. The results showed that fNIRS is sensitive to practice level and mental task load and can monitor hemodynamic changes in the brain.

Bott et al. (12) conducted a Virtual Cognitive Health (VC Health) study in a pre-test and a post-test clinical trial to evaluate its impact on the cognitive function of older adults. Cognitive data function was determined based on the cognitive standardized battery tests that assessed several domains of cognitive function. These tests were administered at baseline, 26 weeks, and 52 weeks, respectively. The data were analyzed to determine the effect of the program on cognitive function and other health outcomes in older adults. The results revealed scalable solutions for the prevention or delay of Alzheimer's disease.

Cammisuli et al. (33) conducted a SENIOR Project that focused on developing technology-based solutions to support the wellbeing of older adults. In this study, the personalized virtual coach platform has two major components: a wearable device/app for patients and a cloud backend server for health professionals. Chan et al. (28) investigated a pre-test/post-test experimental design on iPad training, wherein older adults' cognitive function was measured both before and after the training program. Participants were randomly assigned to either a training group (8 h of training over 4 weeks) or a control group (who did not receive any training). They were measured using several standardized tests for cognitive function, such as memory, attention, and executive function. The results showed that iPad training for older adults can enhance their cognitive function.

Hossain et al. (17) developed a machine learning model to detect CI and analyze neurophysical and physical data and compared its performance in detecting cognitive impairment. A total of 60 older adults (*n* = 30 with CI and 30 without CI) participated in the study. The participants performed a typing task, which involved typing a paragraph of text with a computer keyboard. The physical activity data were collected using a smartwatch worn by the participants. The machine learning models developed in this study achieved a high level of accuracy, sensitivity, and specificity in predicting the cognitive impairment status. Lazarou et al. (13) developed a novel and intelligent home monitoring system to support the care of older adults with CI. The system utilizes a combination of sensor technologies and machine learning algorithms to monitor the daily activities of older adults with cognitive impairment and provides personalized care support.

The system employed a wide range of sensors to monitor the ADL of older adults. The collected data from these sensors were analyzed using machine learning algorithms that helped in identifying patterns and detecting any changes in behavior that may indicate a cognitive decline. Lim et al. (34) employed a hospital-based Internet of Things (IoT) to assess the activities of daily living (ADL) and used it as a digital biomarker to detect CI in a clinical setting. The study employed the experimental ADL test and involved 30 older adults (15 with mild CI and 15 with dementia). The patient's cognitive status was evaluated using the Clinical Dementia Rating (CDR) scale, and the ADL test scores were compared to the patient's cognitive status. Cotelli et al. (29) conducted a study to determine the effect of transcranial magnetic stimulation (TMS) on patients with Alzheimer's disease (AD). This study included 16 patients with AD and 16 healthy control individuals. All participants underwent a series of language assessments, including the action naming test, to assess their language abilities. The results showed that TMS improved the naming ability in AD patients. Another study was conducted by Oyama et al. (15) on CI using high-performance eye-tracking technology. The eye-tracking setup involved the use of a high-performance eye-tracking system that recorded eye movements. As the participant performed the visual search task, the eye-tracking system recorded their eye movements, including the participant's fixation duration, saccade duration, and the number of fixations. The results of the study showed that the eye-tracking method was able to accurately identify individuals with CI with a high level of sensitivity and specificity.

Wang et al. (16) evaluated the effectiveness of a contact sensor skirt for an anti-collision power wheelchair and its safety. Phase 1: Development and Testing of the Contact Sensor Skirt: The researchers used a power wheelchair and attached the sensor skirt to its base to test its technical feasibility. Phase 2: Evaluation of the Contact Sensor Skirt: A randomized controlled trial was performed with 32 older adults with dementia who were randomly assigned to either the intervention group (using the contact sensor skirt) or the control group (using a standard power wheelchair without the sensor skirt). Then, the groups were further assessed based on subjective perceptions of the wheelchair's safety and effectiveness using mobility and safety assessment, observation, questionnaires, and video recordings. The results revealed that the device improved the mobility and wellbeing of older adults.

Xie et al. (10) conducted a study using wearable sensors to assess the gait and distinguish individuals with amnestic mild cognitive impairment (aMCI) from healthy controls. The participants were asked to wear wearable sensors on their lower back and both ankles for 7 days. The sensors collected data during participants' normal daily activities, such as walking, sitting, standing, and lying down. The collected data were processed to extract gait-related features, such as step count, step length, walking speed, and variability of gait parameters. The results of the study showed that there were significant differences in gait parameters between the aMCI group and the healthy control group, with the aMCI group demonstrating a slower walking speed, shorter stride length, and longer double support time. Zhou et al. (37) employed an IoT monitoring system and deep learning classification to assess Alzheimer's disease by using IoT devices, such as wearable sensors, smartphones, and smart homes. These data include physiological signals, cognitive assessments, and daily activities of the patients. An intelligent diagnosis of AD based on an IoT monitoring system and a deep learning classification method involves data collection, preprocessing, feature extraction, model training, and model evaluation. This approach has the potential to improve the accuracy and efficiency of the diagnosis and management of Alzheimer's disease.

Although the use of monitoring-technology devices is associated with several advantages, such as portability, ease of use, enhanced safety, improved caregiver support, and increased independence, it also raises several ethical and patient privacy-related issues that healthcare providers must be aware of. For example, in order to use monitoring-technology devices such as an iPad in assisted cognitive training (28), devices in smart homes (6), the SENIOR Project (33), SMART devices (30), and keystroke patterns (17) in studies, the patient's informed consent must be obtained. However, obtaining informed consent can be challenging for patients with CI. In addition, the devices can reveal sensitive information concerning a person's mental and emotional state, which raises concerns about the privacy, confidentiality, and security of patient data. Digital devices may inadvertently perpetuate bias and discrimination in healthcare. For example, artificial intelligence (AI) algorithms, IoT monitoring systems, fNIRS, transcranial magnetic stimulation (TMS), and deep learning classification may be trained on biased datasets, leading to incorrect diagnoses or treatments for certain populations. Therefore, it is crucial to consider the ethical issues and patient privacy-related issues in the use of digital devices to maintain patient trust, protect patient autonomy, ensure the safety and confidentiality of patient information, and comply with legal and ethical requirements.

Some limitations should be interpreted with caution. Although the monitoring devices selected by the articles included in this review can be useful for older adults with CI, some of the devices were not rigorously evaluated for their effectiveness. Additionally, studies on monitoring devices for older adults with cognitive impairment have small sample sizes, which limits the generalizability of their findings. Some studies focused on older adults with a specific type of cognitive impairment, such as dementia or AD (6, 11, 12, 37), and have not included a diverse range of participants with different cultural backgrounds or socioeconomic statuses. Other studies have short follow-up periods, which limits our understanding of the long-term effectiveness and impact of monitoring devices (28, 37). Finally, by using the scoping reviews method, the inclusion criteria and search strategies used may not identify all relevant studies, potentially leading to bias. Although the articles were selected based on the JBI criteria, the final selection of the articles and synthesis of the literature were based on the judgment of the researchers. This could have introduced subjectivity and potential bias into the review process. Therefore, other standard selection criteria should be included to minimize subjectivity and potential bias. Therefore, further research is needed to evaluate the effectiveness and generalizability of using monitoring devices among older adults.

## 5. Conclusion

Monitoring technologies, including telerehabilitation, smart aging systems, low-cost indoor activity monitoring systems, and wearable sensors for improving cognitive health (such as novel and intelligent home monitoring systems for care and support, technology-enhanced multi-domain at the home continuum of care programs, high-performance eye-tracking technology, contact sensor skirts, functional near-infrared spectroscopy, Internet of Things monitoring system, transcranial magnetic stimulation, and cognitive application) have shown great potential in improving the care and quality of life for older adults with CI. These devices can also provide healthcare professionals with valuable information concerning the older adult's mental health status and daily activities and alert healthcare professionals in case of emergencies and could reduce the care burden. Thus, there is a need to apply this technology practically in the field to further strengthen the evidence that it could improve the lives of older adults.

## 6. Implication for nursing care management practice

During the past few years, there has been growing interest in the use of monitoring-technology devices for older adults with CI. These technological advancements have a potentially critical role in improving the quality of healthcare services to older adults and in promoting their health and wellbeing. Specifically, they can help in promoting positive healthy behavior among older patients. Various monitoring systems (such as high-performance eye-tracking technology, contact sensor skirts, functional near-infrared spectroscopy, Internet of Things monitoring system, transcranial magnetic stimulation, and cognitive application) can help in monitoring their physical activity, maintaining their independence while living at home, and improving their cognitive status, thus improving their quality of life.

These technological device systems and various sensor tools that were developed claimed to be less invasive and more accurate, as they can gather data and detect changes similar to the way nurses diagnose and monitor the health status and activities of older adults with CI even from a distance. Based on the results from the use of remote monitoring systems and smart home technologies, older adults can receive support in their respective homes and communities, reducing the need for institutional care. Healthcare professionals may apply these technologies to attain promising results, such as feasibility and efficiency. Owing to the integration of technology in the field of nursing care, digital knowledge will be required for healthcare professionals to properly navigate the devices. Healthcare professionals, including nurses, must be trained on how to use and interpret the data provided by the devices to provide appropriate care and support.

The integration of technology in nursing care management may also enhance performance and alleviate the challenges encountered during the care of older adults with cognitive impairment, as it is designed to support nurses that need to time to monitor daily information about the patient. Continuum of care can be made possible since some technological devices can be installed even in the homes of older adults. Virtual health coaching and interventions conducted by nurses can also be made possible, which is a vital part of rehabilitation programs of older patients. More studies that focus on the efficiency and applicability of these developed devices and methods in the practical field should be conducted in the future. Nonetheless, it is essential to note that monitoring-technology devices are not a substitute for human interaction and care management. Healthcare professionals, particularly nurses, should use these devices only as a supplement to traditional care and should continue to prioritize human interaction and patient care in meeting older adults' needs and addressing their concerns. Overall, monitoring-technology devices can provide remarkable benefits to older adults by supporting their independence, mobility, safety, and overall wellbeing. Healthcare providers and nurses should work together to incorporate these technological devices into care plans to achieve optimum quality of functioning.

## Data availability statement

The original contributions presented in the study are included in the article/supplementary material, further inquiries can be directed to the corresponding author.

## Author contributions

Conceptualization, methodology, software validation, formal analysis, investigation, data curation, writing—original draft preparation, writing—review and editing, supervision, and project administration: HAs, AA, HAl, and JA. All authors have read and agreed to the published version of the manuscript.
